# EUS-FNA Biopsies to Guide Precision Medicine in Pancreatic Cancer: Results of a Pilot Study to Identify *KRAS* Wild-Type Tumours for Targeted Therapy

**DOI:** 10.3389/fonc.2021.770022

**Published:** 2021-12-09

**Authors:** Joanne Lundy, Marion Harris, John Zalcberg, Allan Zimet, David Goldstein, Val Gebski, Adina Borsaru, Christopher Desmond, Michael Swan, Brendan J. Jenkins, Daniel Croagh

**Affiliations:** ^1^ Centre for Innate Immunity and Infectious Diseases, Hudson Institute of Medical Research, Clayton, VIC, Australia; ^2^ Department of Molecular and Translational Science, Faculty of Medicine, Nursing and Health Sciences, Monash University, Clayton, VIC, Australia; ^3^ Department of Surgery, Faculty of Medicine, Nursing and Health Sciences, Monash University, Clayton, VIC, Australia; ^4^ Department of Oncology, Faculty of Medicine, Nursing and Health Sciences and School of Clinical Sciences, Monash University, Clayton, VIC, Australia; ^5^ Department of Medical Oncology, Alfred Health, Melbourne, VIC, Australia; ^6^ Public Health and Preventative Medicine, Monash University, Melbourne, VIC, Australia; ^7^ Department of Medical Oncology, Epworth Hospital, Melbourne, VIC, Australia; ^8^ Prince of Wales Clinical School, University of New South Wales, Sydney, NSW, Australia; ^9^ Department of Medical Oncology, Prince of Wales Hospital, Randwick, NSW, Australia; ^10^ National Health and Medical Research Council Clinical Trials Centre, University of Sydney, Camperdown, NSW, Australia; ^11^ Diagnostic Imaging, Monash Health, Melbourne, VIC, Australia; ^12^ Department of Gastroenterology, Monash Health, Melbourne, VIC, Australia; ^13^ Department of Surgery, Epworth Healthcare, Melbourne, VIC, Australia

**Keywords:** pancreatic cancer, endoscopic ultrasound, *KRAS*, molecular analysis, precision medicine

## Abstract

**Background:**

Pancreatic ductal adenocarcinoma (PDAC) is a leading cause of cancer death and lacks effective treatment options. Diagnostic endoscopic ultrasound-guided fine-needle aspiration (EUS-FNA) biopsies represent an appealing source of material for molecular analysis to inform targeted therapy, as they are often the only available tissue for patients presenting with PDAC irrespective of disease stage. However, EUS-FNA biopsies are typically not used to screen for precision medicine studies due to concerns about low tissue yield and quality. Epidermal growth factor receptor (EGFR) inhibition has shown promise in clinical trials of unselected patients with advanced pancreatic cancer, but has not been prospectively tested in *KRAS* wild-type patients. Here, we examine the clinical utility of EUS-FNA biopsies for molecular screening of *KRAS* wild-type PDAC patients for targeted anti-EGFR therapy to assess the feasibility of this approach.

**Patients and Methods:**

Fresh frozen EUS-FNA or surgical biopsies from PDAC patient tumours were used to screen for *KRAS* mutations. Eligible patients with recurrent, locally advanced, or metastatic *KRAS* wild-type status who had received at least one prior line of chemotherapy were enrolled in a pilot study (ACTRN12617000540314) and treated with panitumumab at 6mg/kg intravenously every 2 weeks until progression or unacceptable toxicity. The primary endpoint was 4-month progression-free survival (PFS).

**Results:**

275 patient biopsies were screened for *KRAS* mutations, which were detected in 88.3% of patient samples. 8 eligible *KRAS* wild-type patients were enrolled onto the interventional study between November 2017 and December 2020 and treated with panitumumab. 4-month PFS was 14.3% with no objective tumour responses observed. The only grade 3/4 treatment related toxicity observed was hypomagnesaemia.

**Conclusions:**

This study demonstrates proof-of-principle feasibility to molecularly screen patients with pancreatic cancer for targeted therapies, and confirms diagnostic EUS-FNA biopsies as a reliable source of tumour material for molecular analysis. Single agent panitumumab was safe and tolerable but led to no objective tumour responses in this population.

## Introduction

Pancreatic ductal adenocarcinoma (PDAC) ranks as the seventh most lethal cancer worldwide but has been predicted to become the second leading cause of cancer death by 2030 (1, 2). Most patients present with advanced disease, and only 15-20% of tumours are amenable to surgery (3). While the incidence of PDAC continues to steadily increase, the prognosis remains extremely poor with a 5-year survival rate of just 10% (4). For the majority of patients who have unresectable or metastatic disease at diagnosis, treatment options are limited, and median survival is just 6-12 months (5).

Gemcitabine plus nab-paclitaxel and FOLFIRINOX are well established as the first line chemotherapy regimens of choice in patients with advanced PDAC with a good performance status, leading to median survival times of 9-11 months (6, 7). However, recommendations for treatment beyond first line therapy are limited by only a select few phase III clinical trials demonstrating clinical benefit in this setting, and limited head-to-head comparisons using current standards of care (8, 9).

Given the modest effect of chemotherapy in unselected PDAC patients, the prospect of applying precision therapy based on molecular profiling holds great appeal. Unfortunately, clinical trials of targeted therapies in PDAC to date have proved challenging, due to both patient factors (e.g. poor performance status, propensity to rapid clinical deterioration) and practical factors (e.g. poor quality biopsy specimens, delays in processing tissue for molecular analysis, and reliance on surgical biopsies which are not available in the majority of patients) (10, 11). A 2011 review in the US estimated that only 4.5% of patients with PDAC enrolled onto a clinical trial in that year, and identified poor study design, inadequate recruitment, lack of access to suitable trials, and patient factors impeding eligibility to clinical trials as potential barriers to inclusion (12). However, clinical benefit can be demonstrated if actionable molecular alterations are identified and treated with appropriate therapies, such as poly ADP ribose polymerase (PARP) inhibitors for *BRCA* mutant tumours (13).

To overcome the challenges of profiling the molecular and genomic landscape of PDAC in patients from all tumour stages (i.e. I-IV), we have demonstrated the feasibility of endoscopic ultrasound-guided fine needle aspiration (EUS-FNA) biopsy, a common diagnostic procedure, as a reliable source of tissue for genetic profiling (e.g. *KRAS* mutation analysis) (14–16). EUS-FNA using 19 to 25-guage needles is a long-established technique in the diagnosis and staging of pancreatic tumours, and a number of studies have investigated technical aspects to improve the diagnostic performance of the procedure (17, 18). The first generation of FNA biopsy needles provide aspirates of suspicious lesions but often yield lowly cellular specimens lacking in architectural tissue structure, which may be critical for diagnosis as well as for the increasingly desired immunohistochemical and genomic analysis of pancreatic tumours (19, 20). However, newer generation needles allow for larger tissue cores, and have been demonstrated to require fewer needle passes to establish a diagnosis (21, 22). This yields higher volume biopsies with less blood contamination than standard FNA biopsies (23), paving the way for EUS-derived biopsies to play a larger role in molecular profiling in PDAC.

PDAC is typified by significant molecular heterogeneity, and most “actionable” phenotypes such as microsatellite instability, high tumour mutation burden, *BRCA* mutations and *NTRK* fusions occur at a low frequency (24, 25). By contrast, activating mutations of the *KRAS* proto-oncogene can be identified in approximately 80-90% of PDAC patient tumours (24–28). This gene is the focus of ongoing interest as a molecular target for therapy, and although early trials targeting mutant *KRAS* failed to demonstrate any significant survival benefit (29, 30), novel inhibitors are showing more promise in ongoing studies (31).

Epidermal growth factor receptor (EGFR) inhibition has proven to be an effective therapy in *KRAS* wild-type patients with advanced colorectal cancer (32–34). In unselected PDAC patients, the addition of the EGFR inhibitor erlotinib to gemcitabine demonstrated a statistically significant, albeit small improvement in median overall survival (mOS) of 6.3 vs 5.9 months compared to gemcitabine alone (35). However, this finding did not significantly change clinical practice based on minimal benefit and additional toxicity in the erlotinib arm (35). Further studies have reported mixed results using EGFR inhibition in PDAC, albeit largely either without stratification for *KRAS* status, or with *post-hoc* analyses only (35–39). Panitumumab is a recombinant human IgG2 monoclonal antibody that binds specifically to EGFR and has demonstrated clinical efficacy in colorectal cancer (33). In PDAC, a small phase II study with panitumumab, erlotinib and gemcitabine reported a non-significant increase in overall survival in the first line setting when compared to gemcitabine and Erlotinib for PDAC patients who were not selected by *KRAS* status; however, this combination was associated with significant toxicity and limited by the inclusion of a control arm which is no longer considered the standard of care (40).

Here, we aim to demonstrate that standard-of-care diagnostic biopsies sourced from a large PDAC biobank could be used for timely and accurate assessment of *KRAS* mutation status. Specifically, we identified 8 PDAC patients for enrollment onto a pilot study on the efficacy and tolerability of single agent panitumumab for patients with advanced, *KRAS* wild-type PDAC with progressive disease following first line chemotherapy. To the best of our knowledge, this is one of the first prospective biomarker selected studies to date in PDAC.

## Patients and Methods

### Overall Study Design and Ethics Oversight

This study was designed to verify the clinical utility of EUS-FNA biopsies for molecular screening for targeted therapy, and included an exploratory pilot study of single agent panitumumab in patients with *KRAS* wild-type pancreatic cancer. The overall study aims were:

- To show that treatment selection *via* genomic sequencing is feasible in a typical clinical setting.
- To prospectively identify the prevalence of *KRAS* mutations in patients with locally advanced or metastatic pancreatic adenocarcinoma using standard clinical pathology assays.
- To obtain preliminary data on the efficacy of panitumumab in patients with *KRAS* wild-type tumours.

**Figure 1:**
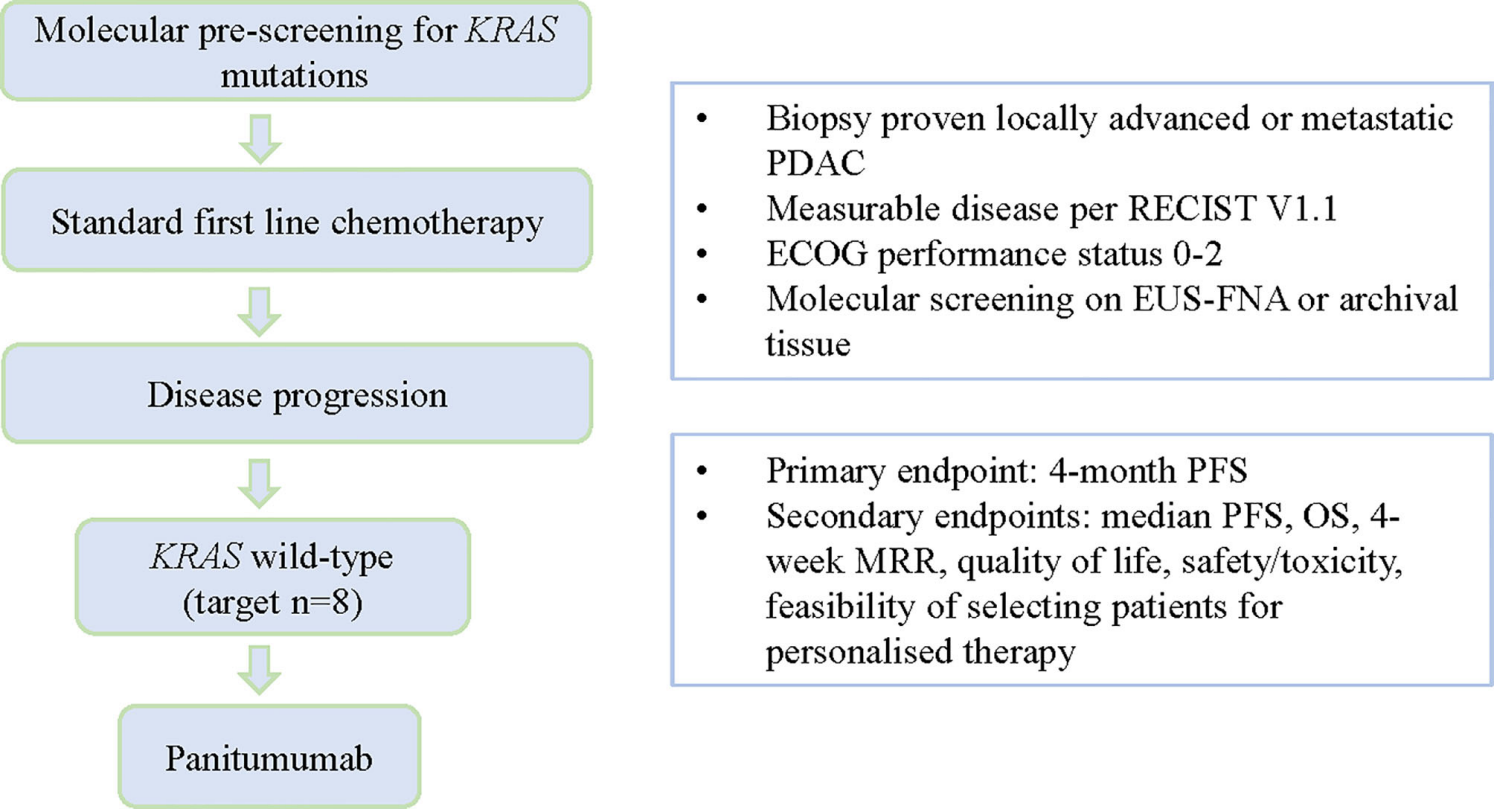
Study schema including key eligibility and response criteria.

The study was conducted in accordance with the principles of the Declaration of Helsinki. It was approved by the Human Research Ethics Committee at Monash Health (reference number 16-0000-584A) and prospectively registered on the Australian New Zealand Clinical Trials Registry (ACTRN12617000540314). Informed, signed written consent was obtained from all patients prior to initiating study procedures. The study schema is shown in **Figure 1**.

### EUS-FNA Biopsies

In addition to the standard diagnostic EUS-FNA biopsy (typically 2-4 needle passes), an additional 1-2 needle passes were employed to obtain tissue for biobanking in the Victorian Pancreatic Cancer Biobank (VPCB; Monash Health HREC reference 15450A). EUS-FNA procedures were carried out in accordance with routine local protocols and needle selection, number of biopsies taken, and suction techniques were at the discretion of each individual physician. The needle type utilised in each case was not recorded as part of this study, but the standard-of-care during the majority of the collection period for this study were the 20 or 22-guage ProCore^®^ Fine Needle Biopsy needles with 10ml negative pressure suction.

### *KRAS* Screening

*KRAS* mutation analysis was performed in the Genetics and Molecular Pathology Department at Monash Health, using the clinically validated *KRAS* StripAssay™ (ViennaLab Diagnostics) in accordance with manufacturer protocols and standard clinical practice. Where possible, DNA was extracted from fresh frozen EUS-FNA biopsies sourced from the multi-centre VPCB using the AllPrep DNA/RNA Universal Kit (Qiagen), although archival formalin-fixed paraffin embedded (FFPE) tissue was used where fresh frozen biopsy tissue was not available. The isolation of gDNA from FFPE tissue was performed on 5 x 10 micron-thick sections using the ReliaPrep FFPE gDNA Miniprep System (Promega). Prospective tissue testing on diagnostic biopsies was preferred, although archival or previously stored specimens from the VPCB could be used where fresh tissue was not feasible or available. DNA samples were quantified using the Qubit Fluorometer (Life Technologies) and quality assessed by TapeStation (Agilent Technologies). At the time of consent to sample collection for the VPCB, patients could elect for their treating physician to be contacted in the event of a significant genomic finding. *KRAS* wild-type results were notified to treating physicians who were able to offer referral for screening for the study if they deemed it clinically appropriate.

### Panitumumab Pilot Study

#### Patient Selection

Patients were eligible if aged 18 and over, with pathologically-proven unresectable, recurrent or metastatic *KRAS* wild-type PDAC (note that patients with pancreaticobiliary type ampullary tumours may be considered on an individual basis, provided they met all other inclusion criteria); ECOG performance status of 0-2, measurable disease as per RECIST v1.1 criteria; progressive disease following at least one line of chemotherapy (defined as either clear progressive disease on standard CT scans or an increase of CA 19.9 of 30% confirmed on 2 blood draws) or within 12 months of adjuvant chemotherapy; adequate bone marrow function (ANC ≥1500/mcL, platelets ≥100 000/mcL, haemoglobin ≥9g/dL); adequate renal function (CrCl > 50ml/min (Cockcroft-Gault formula) or Creatinine <1.5 XULN); and adequate hepatic function (serum total bilirubin ≤ 1.5 times ULN, ALT/AST ≤ 2.5 times ULN [or ≤ 5 times ULN with documented liver metastases], ALP ≤ 5 times ULN, and INR ≤ 1.5). Exclusion criteria included pregnancy or lactation; active or uncontrolled infection; previous treatment with EGFR inhibitor; previous radiotherapy to the pancreas if the only site of measurable disease (unless there was demonstrated, clear evidence of radiological progression at the site since the completion of radiotherapy); hypersensitivity to study drug; previous or current interstitial lung disease or pulmonary fibrosis; history of another malignancy within 2 years prior to allocation (with the exception of adequately treated carcinoma *in-situ*; curatively treated uterine cervix carcinoma *in-situ* or non-melanoma skin carcinoma or superficial transitional cell carcinoma of the bladder); or any severe or uncontrolled medical conditions within 3 months prior to allocation.

#### Study Assessments

Screening procedures and study allocation was independently verified by the principal investigator prior to commencement of study therapy. History, physical examination, assessment of adverse events using NCI CTCAE version 4.0, assessment of ECOG performance status, and routine bloods (FBE, EUC, Ca/Mg/phosphate, LFTs and CA 19.9) were performed at screening and before each treatment with panitumumab. Quality of life was assessed at baseline, week 4, week 16 and at the end of study using the EORTC QLQ-C30 version 3.0 questionnaire, with scores calculated using the EORTC QLC-30 Scoring Manual (41). Serum was collected and stored for assessment of circulating tumour DNA (ctDNA) at baseline, 4 and 8 weeks. An FDG-PET scan was performed at baseline and week 4 to assess for early metabolic response, and to identify patients progressing rapidly for whom alternative treatments should be considered. CT or MRI scans of the chest, abdomen, and pelvis were performed every 8 weeks while on treatment and evaluated for tumour response according to RECIST criteria version 1.1. Treatment was stopped if there was evidence of progressive disease, or at any time according to the discretion of the treating clinician and patient. A 30-day safety assessment was performed at the end of treatment.

#### Treatment

Panitumumab was supplied by Amgen Australia and administered at a standard dose of 6mg/kg by intravenous infusion every 2 weeks. Patients received up to 8 cycles, with the option to continue at the treating physician’s discretion if there was evidence of clinical response. Premedication and supportive care were provided in accordance with local institution protocols, with prophylactic antibiotic therapy strongly recommended to reduce the incidence and severity of rash.

#### Statistical Considerations

This trial was designed as a pilot phase II study, aiming to screen 200 patients to identify the initial cohort of *KRAS* wild-type patients, anticipating a *KRAS* mutation rate of 80-90% and recognizing that some patients would not meet eligibility criteria for the interventional study on the basis of other clinical factors (e.g. poor performance status, inadequate laboratory parameters, clinical decline or death prior to initiation of second line therapy). We deemed that a 50% progression-free survival (PFS) rate at 4 months was considered worthwhile to demonstrate activity of panitumumab, and we planned to enroll 8 patients prior to conducting an assessment to rule out futility (defined as all 8 patients progressing within 4 months, and no metabolic responses seen). In the absence of meeting these criteria, a decision could be made to continue the study and recruit a further 11 patients in a stepwise fashion, guided by the strength of the PFS at 4 months.

The original study design included an observation arm for patients with *KRAS* mutant PDAC receiving physician’s choice standard second line chemotherapy. However, the protocol was amended early in the study to remove this arm due to poor recruitment and lack of perceived benefit of this arm given the small sample size. No patients were recruited onto the initially planned observation cohort prior to the decision to remove this arm.

#### Study Endpoints

The primary endpoint of the interventional study was PFS at 4 months, with secondary endpoints including 4-week metabolic response rate (MMR; defined as a 30% reduction in SUV max on FDG-PET imaging and/or a 30% reduction in CA19.9 if the FDG-PET scan was not abnormal at baseline and the CA19.9 level was elevated >50% above ULN at baseline); PFS and objective tumour response rate (OTRR) at 6 months; feasibility of selecting patients for personalised therapy; median PFS; median overall survival (OS); safety/toxicity; and quality of life. Exploratory endpoints included measurement of ctDNA at baseline and during therapy.

#### Monitoring

A trial management committee including study investigators and a statistician was appointed to oversee study planning, monitoring, progress, reviews, and internal audits.

## Results

### *KRAS* Screening

**Figure 2:**
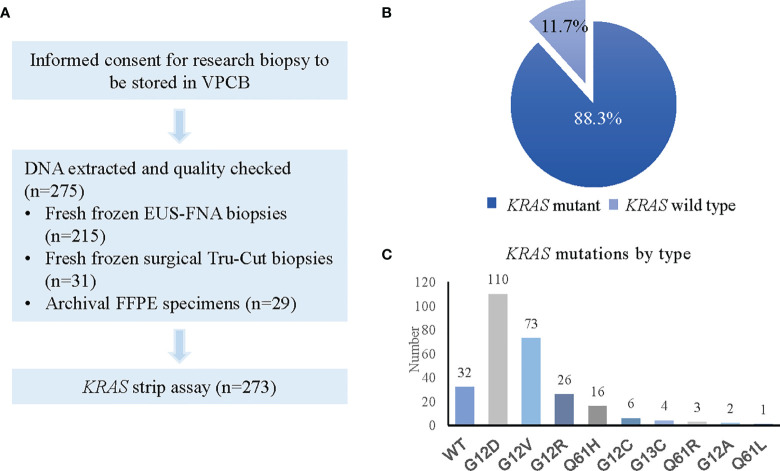
*KRAS* status screening in PDAC patients. **(A)** Flow chart showing process for *KRAS* screening. **(B)** Pie chart representing *KRAS* positivity rate. **(C)** Graph depicting types of *KRAS* mutations detected.

We screened 275 PDAC patient tumour biopsies for the presence of *KRAS* mutations. One fresh frozen EUS-FNA and one FFPE biopsy (0.7%) were deemed inadequate for testing due to poor DNA yield, but all 273 other specimens passed quality control testing. The results of the *KRAS* screening are outlined in **Figure 2**. As anticipated based on existing literature (24–28), *KRAS* was detected in 88.3% (241/273) of tumour biopsies, with 32 samples (11.7%) being wild-type. Frozen FNA, frozen surgical biopsies and FFPE-derived surgical biopsy tissues all demonstrated high frequencies of *KRAS* mutation (88.8%, 93.5% and 89.5%, respectively) with poorer results in the very small number of FFPE-derived EUS-FNA biopsies (**Table 1**). Of the 32 results for *KRAS* wild-type status, 24 patients were deemed ineligible due to physician opinion, performance status, rapid disease progression or death. The remaining 8 patients were enrolled onto study treatment.

**Table 1:** *KRAS* mutation rates in fresh frozen EUS-FNA, surgical and FFPE tissue specimens.

Tissue type	*KRAS* wild-type	*KRAS* mutant
Frozen EUS-FNA biopsy	24 (11.2%)	190 (88.8%)
Frozen surgical biopsy	2 (6.5%)	29 (93.5%)
FFPE tissue		
EUS-FNA biopsy	4 (44.4%)	5 (55.5%)
Surgical specimen	2 (10.5%)	17 (89.5%)

### Patient Characteristics

Between November 2017 and October 2020, 8 patients were enrolled onto the study and treated with panitumumab. The baseline characteristics of the study participants are summarised in **Table 2**. Patients were predominantly male (75%) with an ECOG performance status of 0 or 1 (87.5%) and most had received first line gemcitabine/nab-paclitaxel chemotherapy (62.5%). One patient (12%) had previously undergone a surgical resection and progressed within 12 months of adjuvant gemcitabine/capecitabine. One further patient (12%) had a histological diagnosis of metastatic pancreatic cancer after resection of a previous pancreaticobiliary type tumour of the pancreatic head which was thought to have arisen in the ampulla. After consideration by the principal investigator, this patient was deemed to meet entry criteria for the study. The median time from diagnosis of cancer to enrolment onto this study was 66.2 weeks.

**Table 2:** Baseline patient characteristics.

Characteristic	Frequency (n=8)
Median age in years (range)	64.5 (51-79)
Sex (%)	
Male	6 (75)
Female	2 (25)
Baseline ECOG (%)	
0	2 (25)
1	5 (62.5)
2	1 (12.5)
TNM stage (%)	
III	2 (75)
IV	6 (75)
Previous systemic therapy (%)	
Gemcitabine	2 (25)
Gemcitabine/nab-paclitaxel	5 (62.5)
Gemcitabine/capecitabine	1 (12.5)
FOLFIRINOX	1 (12.5)
Number of metastatic sites (%)	
0	2 (25)
1	3 (37.5)
≥2	3 (37.5)
Site of metastases (%)	
Liver	4 (50)
Lung	4 (50)
Bone	2 (25)
Lymph nodes	2 (25)
CA 19.9 (%)	
<ULN	2 (25)
>ULN	6 (75)
Median time in weeks from initial diagnosis (range)	66.2 (31.2-308.3)

ECOG, Eastern Oncology Cooperative Group; TNM, Tumour Node Metastasis; ULN, upper limit of normal.

### Treatment

Patients received a median of 6 cycles of panitumumab (range 2-9). Seven patients (87.5%) were taken off study treatment due to progressive disease. One patient was taken off study treatment after 6 cycles despite RECIST stable disease, after developing acute urinary retention which led to an unexpected diagnosis of comorbid metastatic prostate cancer. A PSMA-PET scan revealed that the biopsy-confirmed locally advanced pancreatic cancer diagnosed three years prior harboured different metabolic expression compared to the metastatic lesions in the liver and bones, which were consistent with the separate pathology of prostate cancer (also subsequently biopsy proven). This patient was considered not evaluable for response but included in analyses of safety and quality of life.

### Response Measures

**Figure 3:**
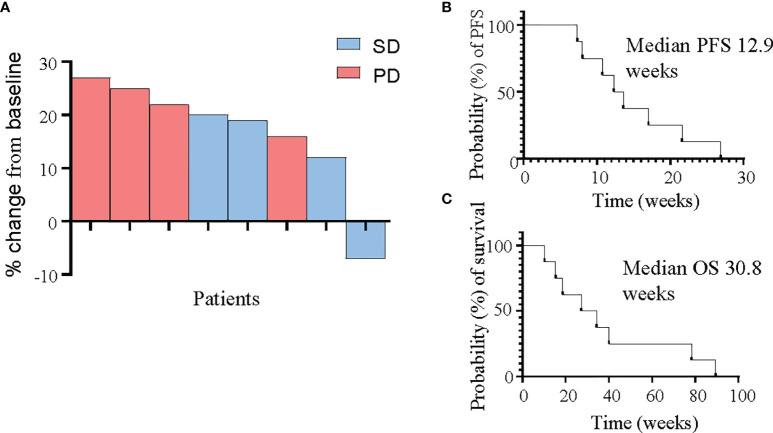
Response of PDAC patients to panitumumab therapy. **(A)** Waterfall plot demonstrating best tumour response as measured by RECIST v1.1 criteria in all 8 patients. **(B, C)** Kaplan-Meier curves for progression-free survival **(B)** and overall survival **(C)**.

The primary endpoint of PFS at 4 months was 14.3%. No metabolic responses were observed, although one patient was identified as a rapid metabolic progressor at the 4-week FDG-PET scan and taken off study treatment. The best response by RECIST v1.1 criteria was stable disease in 4 patients at the initial 8-week assessment, with no objective tumour responses seen and only one patient demonstrating failure to progress at the 16-week assessment. Median PFS was 12.9 weeks, and median OS was 30.8 weeks (**Figure 3**).

Baseline exploratory analysis of ctDNA included digital droplet PCR screening kit for G12/G13 and Q61 *KRAS* mutations. 7 patients (87.5%) had no detectable *KRAS* in the blood; however, the patient who was taken off study early due to rapid clinical and metabolic progression was unexpectedly found to harbour high level *KRAS* mutant allele fraction in their baseline blood sample.

### Safety

Panitumumab was generally well tolerated, in keeping with previous clinical reporting (33). Treatment related adverse events (AEs) are summarised in **Table 3**. The most common treatment related AE was a grade 1 or 2 acneiform rash, occurring in 6/8 patients (75%) and manageable with supportive care. No unexpected or serious drug related toxicity was observed and there were no dose reductions or delays due to toxicity.

**Table 3:** All treatment related adverse events in 8 patients, according to NCI-CTCAE V4.0 criteria.

AE (related)	G1	G2	G3	G4
Fatigue	2	0	0	0
Acneiform rash	6	2	0	0
Anorexia	2	0	0	0
Diarrhoea	1	1	0	0
Pruritis/dry skin	1	0	0	0
Hypomagnesaemia	0	1	1	0
Hand-foot syndrome	2	0	0	0

### Quality of Life

**Figure 4:**
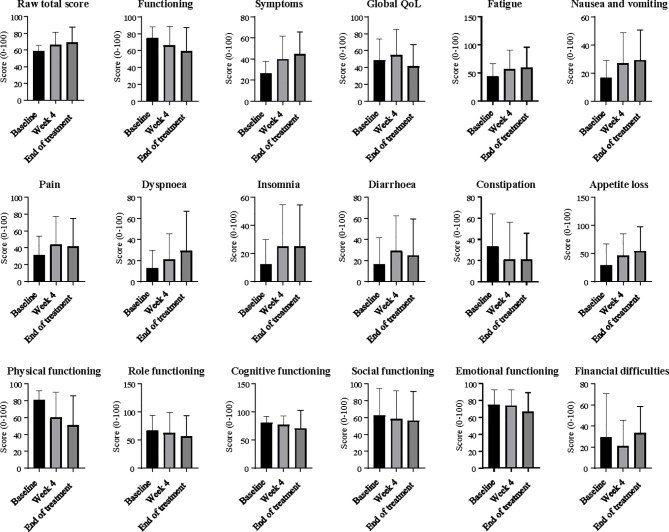
Quality of life scores at baseline, week 4 and end of treatment. Ordinary one-way ANOVA and Tukey’s multiple comparisons test were used to compare differences between timepoints. Error bars on column graphs represent the standard deviation from the mean. No significant changes from baseline were seen in any scores.

Quality of life questionnaires were employed to assess patient-reported outcome measures during treatment. There were no significant changes observed in total raw quality of life scores, or in calculated global quality of life, functioning, or symptom scores between any of the timepoints recorded (**Figure 4**).

## Discussion

This study provides proof-of-principle evidence that EUS-FNA biopsies can be utilised as a source of reliable genetic material to guide timely screening for precision medicine studies in PDAC. The challenges of precision medicine studies in PDAC are well documented, with previous molecularly screened studies failing to achieve recruitment targets due at least in part to tumour specimen inadequacy and processing delays in a patient population requiring timely treatment (10). Here, we demonstrate that selective molecular analysis of EUS-FNA biopsies is sensitive and feasible for patient selection for targeted therapy. Importantly, our reported *KRAS* mutation detection rate was comparable to previous studies which have largely relied on surgical specimens for molecular analysis (24–28). As the majority of patients presenting with PDAC will not undergo surgery, maximizing the use of diagnostic EUS-FNA biopsies for molecular screening and clinical trial selection holds great appeal.

While other groups have reported on EUS-FNA for the isolation of genetic material in PDAC, the methodology, selection criteria for “adequate” samples, and sensitivity of *KRAS* analysis has varied widely. One meta-analysis (15) pooling 931 patients across 9 studies evaluating the role of *KRAS* mutation analysis to improve the diagnostic sensitivity of EUS-FNA in PDAC demonstrated significant heterogeneity across studies and reported a pooled sensitivity rate for the detection of *KRAS* mutations in PDAC of only 76.8% and specificity of 93.3%, figures which fall below acceptable standards to use for therapeutic selection in the clinic. Recent studies of precision medicine in PDAC have either completely excluded EUS-FNA biopsies [e.g. IMPaCT (10), COMPASS (42)] or have failed to report on the success rates of molecular testing in the small number of specimens included [e.g. Know Your Tumour (13)], and the prospective validation of these specimens to guide clinical intervention is not yet established.

We were able to identify and treat 8 patients with *KRAS* wild-type PDAC in our pilot study. While panitumumab had an acceptable safety profile and was generally well tolerated, this study did not meet the predefined primary outcome target of 50% PFS at 4 months. It is difficult to draw definitive conclusions based on our small sample size, particularly with one patient deemed not evaluable for response and one other likely returning a false negative *KRAS* tissue result. However, we elected not to expand the study to include further patients due to slow recruitment, changes in standard second line therapy recommendations since initiating the study (43–46), and increasing availability of alternative molecularly selected studies.Treatment beyond first line chemotherapy in PDAC has been historically challenging. Recently, the final survival analysis of the NAPOLI-1 study demonstrated a significant improvement in median OS (6.1 vs 4,2 months, HR 0.67) with liposomal irinotecan plus 5-FU/leucovorin compared to 5-FU/leucovorin alone in patients previously treated with gemcitabine-based chemotherapy (47), establishing this regimen as standard care in this setting. Prior to this, the CONKO III study had demonstrated significant improvement (4.8 vs 2.3 months) in survival in patients treated with the oxaliplatin/5-FU/folinic acid when compared to best supportive care (BSC), although notably the trial closed early due to poor recruitment and poor acceptance of the BSC arm (48), and the findings were not supported by the subsequent PANCREOX study (49).

While it is not feasible to directly compare survival results between trials, historical standards do provide some benchmarks when considering the results of single arm studies in this setting. A recent meta-analysis of 11 randomised controlled trials of second line therapy including 5-FU and oxaliplatin after first line gemcitabine-based therapy in PDAC reported mOS of 6.3 months in patients with good performance status, with an mOS range of 2.6-6.7 months (50).

We note that despite the lack of clear efficacy of the study drug panitumumab, the mOS in our patient group was reasonably long in comparison to these historic standards (47–50), particularly when considering that the median time from diagnosis to enrolment onto study was over 12 months in our cohort, perhaps reflecting the unique biology and prognosis of *KRAS* wild-type disease in PDAC. While pre-clinical studies including one from our group have demonstrated efficacy of EGFR inhibition in patient-derived xenograft models of *KRAS* wild-type PDAC (14, 51, 52), efforts to translate such promising pre-clinical discoveries to the clinic have often yielded underwhelming results in PDAC (53). This is likely due to a number of contributing factors, including tumour heterogeneity and inadequate pre-clinical modelling of the complex tumour microenvironment and stroma which may hamper drug delivery. Patient-derived organoid models are showing promise in overcoming some of these obstacles, although they remain in their infancy as predictors of real-time clinical response in PDAC (54–57).

*KRAS* has been observed to be a prognostic factor in case series (58) and in *post-hoc* analyses of clinical trials, with a recent meta-analysis including 17 studies of more than 2000 patients reporting a significant association between mutant *KRAS* and overall survival (59). In addition to prognostic significance, it is increasingly clear that *KRAS* wild-type PDAC tumours harbour a distinct clinical and genetic profile when compared to *KRAS* mutant tumours (60, 61). Recently Singhi, et al. reported the results of real-time genome profiling of over 3500 PDAC tumours, including 445 *KRAS* wild-type samples. This study identified potentially targetable genomic changes in 17% of patients, including a number of clinically relevant gene mutations and fusions in the 12% of patients harbouring *KRAS* wild-type tumours (62). In this cohort, 38% of *KRAS* wild-type tumours harboured other alterations with the potential to activate the Receptor Tyrosine Kinase (RTK)/Ras/MAP Kinase (MAPK) signalling pathway, suggesting that more careful selection of patients who may respond to EGFR inhibitor therapy is required beyond *KRAS* alone. *BRAF* alterations have been shown to be mutually exclusive with *KRAS* mutations, and represent another potential therapeutic target in *KRAS* wild-type tumours (62–64). Interestingly, mismatch repair deficits and kinase fusions are also among the genetic changes reported to occur more commonly among patients with a *KRAS* wild-type phenotype (62, 65, 66). While deeper genomic sequencing was outside of the scope of this study, interrogation of our *KRAS* wild-type tumours for other genetic drivers would be extremely valuable in evaluating the lack of response to EGFR inhibition in our patient cohort. It is very likely that some of these patients may have harboured activating mutations in other RTK/Ras/MAPK pathway genes leading to persistent signal activation downstream of EGFR, while others may have had other mechanisms of primary therapeutic resistance. Our findings suggest that *KRAS* alone does not appear to adequately predict for response to EGFR inhibition in PDAC, and we suggest that broader testing of other activating genes in this pathway would be critical in future studies.

We were able to successfully screen almost 300 biopsies for *KRAS* mutations, relying largely on an active local biobank program. We predominantly utilised EUS-FNA biopsies to screen as many patients as possible, and despite variability in yield of genetic material across samples, our *KRAS* mutation rate was consistent with other published literature (24–28). We did not perform broader molecular sequencing as part of this study, which may require higher quality and quantity genetic material than is required for this highly sensitive assay of a common oncogene (67).

Notably, exploratory analysis of ctDNA revealed a strong positive *KRAS* mutant allele fraction in the patient who only completed 2 cycles of panitumumab due to rapid clinical progression, strongly suggesting that the *KRAS* tissue result was a false negative. The *KRAS* StripAssay™ has high sensitivity to detect mutant *KRAS* occurring in 1-5% of tumour cells, and the discordance between tissue and ctDNA testing is difficult to explain given that the tissue testing for this patient was performed on an archival tissue sample containing 80% tumour cellularity. Our exploratory analysis of ctDNA requires further validation in a larger patient cohort, although to date the concordance between tissue and ctDNA findings, and the specificity of the *KRAS* assay used here appear very high (68), suggesting that the patient in this study was an outlier. However, this finding highlights the need to optimize the accuracy of molecular testing in PDAC, and lends weight to an argument to incorporate liquid biopsies into clinical trial design as another layer of molecular screening to enhance sensitivity and specificity. Our study was performed in a “real-world” setting using standard-of-care biopsies at the discretion of each individual physician, and we did not collect information about the specific types of needles and techniques used to collect the EUS-FNA biopsies. With advancements in technology, the newer-generation core biopsy needles are becoming more commonplace in routine medical practice and in research, and are likely to improve yield and quality of the samples used for genomic analysis. In future studies, we plan to explore the diagnostic accuracy, yield and quality of genetic material obtained with newer generation needles (e.g. Acquire™ or SharkCore™).

EGFR inhibition is not a novel concept in the treatment of PDAC, although results have been mixed. A phase II study of the anti-EGFR monoclonal antibody nimotuzumab in combination with gemcitabine recently demonstrated activity and tolerability in the first line setting, most markedly in *KRAS* wild-type patients. However, the *KRAS* mutation analysis was again performed retrospectively, only available in approximately 50% of participants, and positive in far fewer than expected (37). A previous meta-analysis of 4 randomised control trials of cetuximab revealed no survival benefit, but significant additional toxicity (39), while combined meta-analysis of 24 studies including erlotinib with gemcitabine reported modest evidence of efficacy but did not explore molecular subgroups (38). Notably, a recent systematic review of phase II trials in advanced PDAC reported that of 37 trials investigating biologic agents, just 1 included prospective biomarker enrichment (69). *Post-hoc* analyses have reported conflicting findings with regards to the predictive value of *KRAS* for EGFR pathway inhibition, but are often hampered by incomplete genomic information for study cohorts and lower than expected *KRAS* mutation rates, suggesting the presence of false negative results (36, 60, 70–72).

This study was not designed or powered to detect a benefit of panitumumab over other standard of care agents in PDAC which would require a large multicentre study given the rarity of *KRAS* wild-type tumours and well documented challenges in enrolling patients with PDAC onto clinical trials. We primarily aimed to demonstrate proof-of-principal evidence for routine use of EUS-FNA derived material in real-time molecular screening in PDAC, with a secondary aim to detect preliminary signals of panitumumab efficacy which could be used to justify larger subsequent expansion of the study. Despite the small sample size, we saw no objective responses to therapy, and when examined in the context of previous studies, our study does not offer any convincing evidence that panitumumab demonstrates adequate efficacy to pursue further in this setting.

PDAC is an aggressive malignancy associated with rapid clinical decline, and optimal clinical trial design needs to be carefully considered in this setting. The recent encouraging results of the POLO study demonstrated that maintenance therapy is feasible in this patient population (46). A maintenance approach to anti-EGFR targeted therapy for PDAC patients without *KRAS* or other activating MAPK pathway mutations who achieve disease control on first line chemotherapy may be worthy of exploration in future studies.

As our understanding of the molecular landscape of PDAC has expanded, and with the recent demonstration of a PFS benefit in patients harbouring germline *BRCA* mutations undergoing maintenance olaparib therapy after failure to progress on platinum-based chemotherapy (46), molecular testing is now routinely recommended in several therapeutic guidelines (8, 73). More recently, several ongoing studies have encouragingly begun to report successful implementation of molecularly matched therapies in PDAC (13, 42), providing ongoing hope for the expansion of precision medicine to improve patient outcomes.

## Conclusion

In summary, our study confirms that rapid, prospective molecular testing of EUS-FNA diagnostic biopsies can accurately detect *KRAS* mutations in PDAC, and is among the first to prospectively enroll molecularly screened patients onto targeted therapy using EUS-FNA biopsies. Notably, our findings provide key evidence that precision medicine in PDAC can be feasibly applied in clinical trials in the ongoing endeavor to improve outcomes in this deadly disease, and lay the foundation for the continual refinement of targeted therapy approaches in PDAC. Furthermore, our pilot study of single agent panitumumab proved safe and tolerable, but showed no significant signal of efficacy in patients with advanced *KRAS* wild-type PDAC treated after standard chemotherapy. Median survival in the *KRAS* wild-type patient group was longer than historical controls, in keeping with reports from other groups. Importantly, EUS-FNA biopsies proved a feasible source of tissue for rapid *KRAS* analysis of large numbers of patients and highlighted the value of tissue biobanking and the potential utility of these often low-yield biopsies to increase patient access to molecular testing and matched therapies in future studies.

## Data Availability Statement

The raw data supporting the conclusions of this article will be made available by the authors, without undue reservation.

## Ethics Statement

The studies involving human participants were reviewed and approved by Human Research Ethics Committee at Monash Health. The patients/participants provided their written informed consent to participate in this study.

## Author Contributions

Study conception and design: DC, JZ, DG, AZ, VG, and JL. Human tissue acquisition: DC, CD, and MS. Acquisition of study data: DC, MH, and JL. Analysis and interpretation of data: JL, DC, MH, JZ, AZ, DG, VG, AB, and BJ. Writing and/or revision of the manuscript: JL, DC, MH, JZ, AZ, DG, VG, and BJ. Study supervision: DC and BJ. All authors contributed to the article and approved the submitted version.

## Funding

This study was supported by a Monash Partners Comprehensive Cancer Consortium (MPCCC) Research Grant and an Epworth Medical Foundation (EMF) research grant. Additional funding was obtained from Amgen, who also supplied the study drug, and the Operational Infrastructure Support Program by the Victorian Government of Australia. BJJ is supported by a Senior Research Fellowship from the National Health and Medical Research Council of Australia.

## Conflict of Interest

The authors declare that the research was conducted in the absence of any commercial or financial relationships that could be construed as a potential conflict of interest.

This study received partial funding from Amgen. The funder had the following involvement with the study: review of study design, provision of study drug and partial funding for study procedures.

The handling editor SS and the reviewer EC have declared a shared parent affiliation with the author VG at the time of review.

## Publisher’s Note

All claims expressed in this article are solely those of the authors and do not necessarily represent those of their affiliated organizations, or those of the publisher, the editors and the reviewers. Any product that may be evaluated in this article, or claim that may be made by its manufacturer, is not guaranteed or endorsed by the publisher.

## References

[1] Sung H Ferlay J Siegel RL Laversanne M Soerjomataram I Jemal A. Global Cancer Statistics 2020: GLOBOCAN Estimates of Incidence and Mortality Worldwide for 36 Cancers in 185 Countries. CA Cancer J Clin (2021) 71(3):209–49. doi: 10.3322/caac.21660 33538338

[2] Rahib L Smith BD Aizenberg R Rosenzweig AB Fleshman JM Matrisian LM. Projecting Cancer Incidence and Deaths to 2030: The Unexpected Burden of Thyroid, Liver, and Pancreas Cancers in the United States. Cancer Res (2014) 74:2913. doi: 10.1158/0008-5472.CAN-14-0155 24840647

[3] Wade TP Halaby IA Stapleton DR Virgo KS Johnson FE. Population-Based Analysis of Treatment of Pancreatic Cancer and Whipple Resection: Department of Defense Hospitals, 1989–1994. Surgery (1996) 120:680–7. doi: 10.1016/S0039-6060(96)80017-1 8862378

[4] Siegel RL Miller KD Fuchs HE Jemal A. Cancer Statistics, 2021. CA Cancer J Clin (2021) 71:7–33. doi: 10.3322/caac.21654 33433946

[5] Huang L Jansen L Balavarca Y Babaei M van der Geest L Lemmens V. Stratified Survival of Resected and Overall Pancreatic Cancer Patients in Europe and the USA in the Early Twenty-First Century: A Large, International Population-Based Study. BMC Med (2018) 16:125–5. doi: 10.1186/s12916-018-1120-9 PMC6102804 30126408

[6] Von Hoff DD Ervin T Arena FP Chiorean EG Infante J Moore M. Increased Survival in Pancreatic Cancer With Nab-Paclitaxel Plus Gemcitabine. N Engl J Med (2013) 369:1691–703. doi: 10.1056/NEJMoa1304369 PMC4631139 24131140

[7] Conroy T Desseigne F Ychou M Bouche O Guimbaud R Becouarn Y. FOLFIRINOX Versus Gemcitabine for Metastatic Pancreatic Cancer. N Engl J Med (2011) 364:1817–25. doi: 10.1056/NEJMoa1011923 21561347

[8] Sohal DPS Kennedy EB Cinar P Conroy T Copur MS Crane CH. Metastatic Pancreatic Cancer: ASCO Guideline Update. J Clin Oncol (2020) 38:3217–30. doi: 10.1200/jco.20.01364 PMC12974607 32755482

[9] Ducreux M Cuhna AS Caramella C Hollebecque A Burtin P Goéré D. Cancer of the Pancreas: ESMO Clinical Practice Guidelines for Diagnosis, Treatment and Follow-Up. Ann Oncol (2015) 26 Suppl 5:v56–68. doi: 10.1093/annonc/mdv295 26314780

[10] Chantrill LA Nagrial AM Watson C Johns AL Martyn-Smith M Simpson S. Precision Medicine for Advanced Pancreas Cancer: The Individualized Molecular Pancreatic Cancer Therapy (IMPaCT) Trial. Clin Cancer Res (2015) 21:2029–37. doi: 10.1158/1078-0432.CCR-15-0426 25896973

[11] Ding D Javed AA Cunningham D Teinor J Wright M Javed ZN. Challenges of the Current Precision Medicine Approach for Pancreatic Cancer: A Single Institution Experience Between 2013 and 2017. Cancer Lett (2021) 497:221–8. doi: 10.1016/j.canlet.2020.10.039 PMC8375587 33127389

[12] Hoos WA James PM Rahib L Talley AW Fleshman JM Matrisian LM. Pancreatic Cancer Clinical Trials and Accrual in the United States. J Clin Oncol (2013) 31:3432–8. doi: 10.1200/jco.2013.49.4823 23960185

[13] Pishvaian MJ Bender RJ Halverson D Rahib L Hendifar AE Mikhail S. Molecular Profiling of Patients With Pancreatic Cancer: Initial Results From the Know Your Tumor Initiative. Clin Cancer Res (2018) 24:5018–27. doi: 10.1158/1078-0432.Ccr-18-0531 29954777

[14] Berry W Algar E Kumar B Desmond C Swan M Jenkins BJ. Endoscopic Ultrasound-Guided Fine-Needle Aspirate-Derived Preclinical Pancreatic Cancer Models Reveal Panitumumab Sensitivity in KRAS Wild-Type Tumors. Int J Cancer (2017) 140:2331–43. doi: 10.1002/ijc.30648 28198009

[15] Fuccio L Hassan C Laterza L Correale L Pagano N Bocus P. The Role of K-Ras Gene Mutation Analysis in EUS-Guided FNA Cytology Specimens for the Differential Diagnosis of Pancreatic Solid Masses: A Meta-Analysis of Prospective Studies. Gastrointest Endosc (2013) 78:596–608. doi: 10.1016/j.gie.2013.04.162 23660563

[16] Lundy J Gao H Berry W Masoumi-Moghoddam S Jenkins BJ Croagh D. Targeted Transcriptome and KRAS Mutation Analysis Improve the Diagnostic Performance of EUS-FNA Biopsies in Pancreatic Cancer. Clin Cancer Res (2021) 27(21):5900–11. doi: 10.1158/1078-0432.Ccr-21-1107 34400416

[17] Marques S Bispo M Rio-Tinto R Fidalgo P Devière J. The Impact of Recent Advances in Endoscopic Ultrasound-Guided Tissue Acquisition on the Management of Pancreatic Cancer. GE Port J Gastroenterol (2021) 28:185–92. doi: 10.1159/000510730 PMC8138259 34056041

[18] Polkowski M Jenssen C Kaye P Carrara S Deprez P Gines A. Technical Aspects of Endoscopic Ultrasound (EUS)-Guided Sampling in Gastroenterology: European Society of Gastrointestinal Endoscopy (ESGE) Technical Guideline – March 2017. Endoscopy (2017) 49:989–1006. doi: 10.1055/s-0043-119219 28898917

[19] Khoury T Sbeit W Ludvik N Nadella D Wiles A Marshall C. Concise Review on the Comparative Efficacy of Endoscopic Ultrasound-Guided Fine-Needle Aspiration vs Core Biopsy in Pancreatic Masses, Upper and Lower Gastrointestinal Submucosal Tumors. World J Gastrointest Endosc (2018) 10:267–73. doi: 10.4253/wjge.v10.i10.267 PMC6198315 30364716

[20] James T Baron T. A Comprehensive Review of Endoscopic Ultrasound Core Biopsy Needles. Expert Rev Med Devices (2018) 15:127–135. doi: 10.1080/17434440.2018.1425137 29334842

[21] Alatawi A Beuvon F Grabar S Leblanc S Chaussade S Terris B. Comparison of 22G Reverse-Beveled Versus Standard Needle for Endoscopic Ultrasound-Guided Sampling of Solid Pancreatic Lesions. United European Gastroenterol J (2015) 3:343–52. doi: 10.1177/2050640615577533 PMC4528208 26279842

[22] Bang JY Hawes R Varadarajulu S. A Meta-Analysis Comparing ProCore and Standard Fine-Needle Aspiration Needles for Endoscopic Ultrasound-Guided Tissue Acquisition. Endoscopy (2016) 48:339–49. doi: 10.1055/s-0034-1393354 26561917

[23] Mukai S Itoi T Katanuma A Irisawa A. An Animal Experimental Study to Assess the Core Tissue Acquisition Ability of Endoscopic Ultrasound-Guided Histology Needles. Endosc Ultrasound (2018) 7:263–9. doi: 10.4103/eus.eus_16_17 PMC6106151 28836511

[24] Waddell N Pajic M Patch AM Chang DK Kassahn KS Bailey P. Whole Genomes Redefine the Mutational Landscape of Pancreatic Cancer. Nature (2015) 518:495–501. doi: 10.1038/nature14169 25719666 PMC4523082

[25] Witkiewicz AK McMillan EA Balaji U Baek G Lin WC Mansour J. Whole-Exome Sequencing of Pancreatic Cancer Defines Genetic Diversity and Therapeutic Targets. Nat Commun (2015) 6:6744. doi: 10.1038/ncomms7744 25855536 PMC4403382

[26] Almoguera C Shibata D Forrester K Martin J Arnheim N Perucho M. Most Human Carcinomas of the Exocrine Pancreas Contain Mutant C-K-Ras Genes. Cell (1988) 53:549–54. doi: 10.1016/0092-8674(88)90571-5 2453289

[27] Biankin AV Waddell N Kassahn KS Gingras MC Muthuswamy LB Johns AL. Pancreatic Cancer Genomes Reveal Aberrations in Axon Guidance Pathway Genes. Nature (2012) 491:399–405. doi: 10.1038/nature11547 23103869 PMC3530898

[28] Jones S Zhang X Parsons DW Lin JC Leary RJ Angenendt P. Core Signaling Pathways in Human Pancreatic Cancers Revealed by Global Genomic Analyses. Science (2008) 321:1801–6. doi: 10.1126/science.1164368 PMC2848990 18772397

[29] Macdonald JS McCoy S Whitehead RP Iqbal S Wade JL 3rd Giguere JK. A Phase II Study of Farnesyl Transferase Inhibitor R115777 in Pancreatic Cancer: A Southwest Oncology Group (SWOG 9924) Study. Invest New Drugs (2005) 23:485–7. doi: 10.1007/s10637-005-2908-y 16133800

[30] Marín-Ramos NI Ortega-Gutiérrez S and López-Rodríguez ML. Blocking Ras Inhibition as an Antitumor Strategy. Semin Cancer Biol (2019) 54:91–100. doi: 10.1016/j.semcancer.2018.01.017 29409706

[31] Hong DS Kuo J Sacher AG Barlesi F Besse B Kuboki Y. CodeBreak 100: Phase I Study of AMG 510, a Novel KRASG12C Inhibitor, in Patients (Pts) With Advanced Solid Tumors Other Than Non-Small Cell Lung Cancer (NSCLC) and Colorectal Cancer (CRC). J Clin Oncol (2020) 38:3511–1. doi: 10.1200/JCO.2020.38.15_suppl.3511

[32] Amado RG Wolf M Peeters M Van Cutsem E Siena S Freeman DJ. Wild-Type KRAS Is Required for Panitumumab Efficacy in Patients With Metastatic Colorectal Cancer. J Clin Oncol (2008) 26:1626–34. doi: 10.1200/JCO.2007.14.7116 18316791

[33] Douillard JY Siena S Cassidy J Tabernero J Burkes R Barugel M. Randomized, Phase III Trial of Panitumumab With Infusional Fluorouracil, Leucovorin, and Oxaliplatin (FOLFOX4) Versus FOLFOX4 Alone as First-Line Treatment in Patients With Previously Untreated Metastatic Colorectal Cancer: The PRIME Study. J Clin Oncol (2010) 28:4697–705. doi: 10.1200/jco.2009.27.4860 20921465

[34] Van Cutsem E Köhne CH Láng I Folprecht G Nowacki MP Cascinu S. Cetuximab Plus Irinotecan, Fluorouracil, and Leucovorin as First-Line Treatment for Metastatic Colorectal Cancer: Updated Analysis of Overall Survival According to Tumor KRAS and BRAF Mutation Status. J Clin Oncol (2011) 29:2011–9. doi: 10.1200/jco.2010.33.5091 21502544

[35] Moore MJ Goldstein D Hamm J Figer A Hecht JR Gallinger S. Erlotinib Plus Gemcitabine Compared With Gemcitabine Alone in Patients With Advanced Pancreatic Cancer: A Phase III Trial of the National Cancer Institute of Canada Clinical Trials Group. J Clin Oncol (2007) 25:1960–6. doi: 10.1200/JCO.2006.07.9525 17452677

[36] Boeck S Jung A Laubender RP Neumann J Egg R Goritschan C. EGFR Pathway Biomarkers in Erlotinib-Treated Patients With Advanced Pancreatic Cancer: Translational Results From the Randomised, Crossover Phase 3 Trial AIO-Pk0104. Br J Cancer (2013) 108:469–76. doi: 10.1038/bjc.2012.495 PMC3566829 23169292

[37] Schultheis B Reuter D Ebert MP Siveke J Kerkhoff A Berdel WE. Gemcitabine Combined With the Monoclonal Antibody Nimotuzumab Is an Active First-Line Regimen in KRAS Wildtype Patients With Locally Advanced or Metastatic Pancreatic Cancer: A Multicenter, Randomized Phase IIb Study. Ann Oncol (2017) 28:2429–35. doi: 10.1093/annonc/mdx343 28961832

[38] Wang Y Hu GF Zhang QQ Tang N Guo J Liu LY. Efficacy and Safety of Gemcitabine Plus Erlotinib for Locally Advanced or Metastatic Pancreatic Cancer: A Systematic Review and Meta-Analysis. Drug Des Devel Ther (2016) 10:1961–72. doi: 10.2147/dddt.S105442 PMC4912328 27358556

[39] Forster T Huettner FJ Springfeld C Loehr M Kalkum E Hackbusch M. Cetuximab in Pancreatic Cancer Therapy: A Systematic Review and Meta-Analysis. Oncology (2020) 98:53–60. doi: 10.1159/000502844 31578019

[40] Halfdanarson TR Foster NR Kim GP Meyers JP Smyrk TC McCullough AE. A Phase II Randomized Trial of Panitumumab, Erlotinib, and Gemcitabine Versus Erlotinib and Gemcitabine in Patients With Untreated, Metastatic Pancreatic Adenocarcinoma: North Central Cancer Treatment Group Trial N064B (Alliance). Oncologist (2019) 24:589–e160. doi: 10.1634/theoncologist.2018-0878 30679315 PMC6516109

[41] Aaronson NK Ahmedzai S Bergman B Bullinger M Cull A Duez NJ. The European Organization for Research and Treatment of Cancer QLQ-C30: A Quality-of-Life Instrument for Use in International Clinical Trials in Oncology. J Natl Cancer Inst (1993) 85:365–76. doi: 10.1093/jnci/85.5.365 8433390

[42] Aung KL Fischer SE Denroche RE Jang GH Dodd A Creighton S. Genomics-Driven Precision Medicine for Advanced Pancreatic Cancer: Early Results From the COMPASS Trial. Clin Cancer Res (2018) 24:1344–54. doi: 10.1158/1078-0432.Ccr-17-2994 PMC5968824 29288237

[43] Mita N Iwashita T Uemura S Yoshida K Iwasa Y Ando N. Second-Line Gemcitabine Plus Nab-Paclitaxel for Patients With Unresectable Advanced Pancreatic Cancer After First-Line FOLFIRINOX Failure. J Clin Med (2019) 8(6):761. doi: 10.3390/jcm8060761 PMC6616879 31146420

[44] Portal A Pernot S Tougeron D Arbaud C Bidault AT de la Fouchardière C. Nab-Paclitaxel Plus Gemcitabine for Metastatic Pancreatic Adenocarcinoma After Folfirinox Failure: An AGEO Prospective Multicentre Cohort. Br J Cancer (2015) 113:989–95. doi: 10.1038/bjc.2015.328 PMC4651133 26372701

[45] Wang-Gillam A Li CP Bodoky G Dean A Shan YS Jameson G. Nanoliposomal Irinotecan With Fluorouracil and Folinic Acid in Metastatic Pancreatic Cancer After Previous Gemcitabine-Based Therapy (NAPOLI-1): A Global, Randomised, Open-Label, Phase 3 Trial. Lancet (London England) (2016) 387:545–57. doi: 10.1016/s0140-6736(15)00986-1 26615328

[46] Golan T Hammel P Reni M Van Cutsem E Macarulla T Hall MJ. Maintenance Olaparib for Germline BRCA-Mutated Metastatic Pancreatic Cancer. N Engl J Med (2019) 381:317–27. doi: 10.1056/NEJMoa1903387 PMC6810605 31157963

[47] Wang-Gillam A Hubner RA Siveke JT Von Hoff DD Belanger B de Jong FA. NAPOLI-1 Phase 3 Study of Liposomal Irinotecan in Metastatic Pancreatic Cancer: Final Overall Survival Analysis and Characteristics of Long-Term Survivors. Eur J Cancer (2019) 108:78–87. doi: 10.1016/j.ejca.2018.12.007 30654298

[48] Pelzer U Schwaner I Stieler J Adler M Seraphin J Dörken B. Best Supportive Care (BSC) Versus Oxaliplatin, Folinic Acid and 5-Fluorouracil (OFF) Plus BSC in Patients for Second-Line Advanced Pancreatic Cancer: A Phase III-Study From the German CONKO-Study Group. Eur J Cancer (2011) 47:1676–81. doi: 10.1016/j.ejca.2011.04.011 21565490

[49] Gill S Ko YJ Cripps C Beaudoin A Dhesy-Thind S Zulfiqar M. PANCREOX: A Randomized Phase III Study of Fluorouracil/Leucovorin With or Without Oxaliplatin for Second-Line Advanced Pancreatic Cancer in Patients Who Have Received Gemcitabine-Based Chemotherapy. J Clin Oncol (2016) 34:3914–20. doi: 10.1200/jco.2016.68.5776 27621395

[50] Wainberg ZA Feeney K Lee MA Muñoz A Gracián AC Lonardi S. Meta-Analysis Examining Overall Survival in Patients With Pancreatic Cancer Treated With Second-Line 5-Fluorouracil and Oxaliplatin-Based Therapy After Failing First-Line Gemcitabine-Containing Therapy: Effect of Performance Status and Comparison With Other Regimens. BMC Cancer (2020) 20:633. doi: 10.1186/s12885-020-07110-x 32641104 PMC7346629

[51] Bruns CJ Solorzano CC Harbison MT Ozawa S Tsan R Fan D. Blockade of the Epidermal Growth Factor Receptor Signaling by a Novel Tyrosine Kinase Inhibitor Leads to Apoptosis of Endothelial Cells and Therapy of Human Pancreatic Carcinoma. Cancer Res (2000) 60:2926–35. 10850439

[52] Ng SS Tsao MS Nicklee T Hedley DW. Effects of the Epidermal Growth Factor Receptor Inhibitor OSI-774, Tarceva, on Downstream Signaling Pathways and Apoptosis in Human Pancreatic Adenocarcinoma. Mol Cancer Ther (2002) 1:777–83. 12492110

[53] Lieu CH Tan A-C Leong S Diamond JR Eckhardt SG. From Bench to Bedside: Lessons Learned in Translating Preclinical Studies in Cancer Drug Development. J Natl Cancer Inst (2013) 105:1441–56. doi: 10.1093/jnci/djt209 PMC3787906 24052618

[54] Gutierrez-Barrera AM Menter DG Abbruzzese JL Reddy SA. Establishment of Three-Dimensional Cultures of Human Pancreatic Duct Epithelial Cells. Biochem Biophys Res Commun (2007) 358:698–703. doi: 10.1016/j.bbrc.2007.04.166 17512909 PMC2562614

[55] Tiriac H Belleau P Engle DD Plenker D Deschênes A Somerville TDD. Organoid Profiling Identifies Common Responders to Chemotherapy in Pancreatic Cancer. Cancer Discov (2018) 8:1112–29. doi: 10.1158/2159-8290.Cd-18-0349 PMC6125219 29853643

[56] Pasch CA Favreau PF Yueh AE Babiarz CP Gillette AA Sharick JT. Patient-Derived Cancer Organoid Cultures to Predict Sensitivity to Chemotherapy and Radiation. Clin Cancer Res (2019) 25:5376–87. doi: 10.1158/1078-0432.Ccr-18-3590 PMC6726566 31175091

[57] Frappart P-O Walter K Gout J Beutel AK Morawe M Arnold F. Pancreatic Cancer-Derived Organoids – a Disease Modeling Tool to Predict Drug Response. United European Gastroenterol J (2020) 8:594–606. doi: 10.1177/2050640620905183 PMC7268941 32213029

[58] Windon AL Loaiza-Bonilla A Jensen CE Randall M Morrissette JJD. A KRAS Wild Type Mutational Status Confers a Survival Advantage in Pancreatic Ductal Adenocarcinoma. J Gastrointest Oncol (2018) 9:1–10. doi: 10.21037/jgo.2017.10.14 29564165 PMC5848049

[59] Tao L-Y Zhang L-F Xiu D-R Yuan C-h Ma Z-l Jiang B. Prognostic Significance of K-Ras Mutations in Pancreatic Cancer: A Meta-Analysis. World J Surg Oncol (2016) 14:146. doi: 10.1186/s12957-016-0888-3 27183870 PMC4868030

[60] Kim ST Lim DH Jang KT Lim T Lee J Choi YL. Impact of KRAS Mutations on Clinical Outcomes in Pancreatic Cancer Patients Treated With First-Line Gemcitabine-Based Chemotherapy. Mol Cancer Ther (2011) 10:1993–9. doi: 10.1158/1535-7163.Mct-11-0269 21862683

[61] Luchini C Paolino G Mattiolo P Piredda ML Cavaliere A Gaule M. KRAS Wild-Type Pancreatic Ductal Adenocarcinoma: Molecular Pathology and Therapeutic Opportunities. J Exp Clin Cancer Res (2020) 39:227. doi: 10.1186/s13046-020-01732-6 33115526 PMC7594413

[62] Singhi AD George B Greenbowe JR Chung J Suh J Maitra A. Real-Time Targeted Genome Profile Analysis of Pancreatic Ductal Adenocarcinomas Identifies Genetic Alterations That Might Be Targeted With Existing Drugs or Used as Biomarkers. Gastroenterology (2019) 156:2242–53.e2244. doi: 10.1053/j.gastro.2019.02.037 30836094

[63] Wrzeszczynski KO Rahman S Frank MO Arora K Shah M Geiger H. Identification of Targetable BRAF Δn486_P490 Variant by Whole-Genome Sequencing Leading to Dabrafenib-Induced Remission of a BRAF-Mutant Pancreatic Adenocarcinoma. Mol Case Stud (2019) 5(6). doi: 10.1101/mcs.a004424 PMC6913137 31519698

[64] Del Curatolo A Conciatori F Cesta Incani U Bazzichetto C Falcone I Corbo V. Therapeutic Potential of Combined BRAF/MEK Blockade in BRAF-Wild Type Preclinical Tumor Models. J Exp Clin Cancer Res (2018) 37:140. doi: 10.1186/s13046-018-0820-5 29986755 PMC6038340

[65] Goggins M Offerhaus GJ Hilgers W Griffin CA Shekher M Tang D. Pancreatic Adenocarcinomas With DNA Replication Errors (RER+) Are Associated With Wild-Type K-Ras and Characteristic Histopathology. Poor Differentiation, a Syncytial Growth Pattern, and Pushing Borders Suggest RER+. Am J Pathol (1998) 152:1501–7. PMC1858440 9626054

[66] Luchini C Brosens LAA Wood LD Chatterjee D Shin JI Sciammarella C. Comprehensive Characterisation of Pancreatic Ductal Adenocarcinoma With Microsatellite Instability: Histology, Molecular Pathology and Clinical Implications. Gut (2021) 70:148–56. doi: 10.1136/gutjnl-2020-320726 PMC7211065 32350089

[67] Imaoka H Sasaki M Hashimoto Y Watanabe K Ikeda M. New Era of Endoscopic Ultrasound-Guided Tissue Acquisition: Next-Generation Sequencing by Endoscopic Ultrasound-Guided Sampling for Pancreatic Cancer. J Clin Med (2019) 8:1173. doi: 10.3390/jcm8081173 PMC6723875 31387310

[68] Sellahewa R Lundy J Croagh D Jenkins B. High Circulating Tumour DNA Is a Strong Negative Prognostic Factor in Operable Pancreatic Cancer. HPB (2021) 23:S263. doi: 10.1016/j.hpb.2020.11.664

[69] Tang M Chen J Goldstein D Links M Lord S Marschner I. Evaluation of Phase II Trial Design in Advanced Pancreatic Cancer. Pancreas (2019) 48:1274–84. doi: 10.1097/mpa.0000000000001429 31688590

[70] Infante JR Somer BG Park JO Li CP Scheulen ME Kasubhai SM. A Randomised, Double-Blind, Placebo-Controlled Trial of Trametinib, an Oral MEK Inhibitor, in Combination With Gemcitabine for Patients With Untreated Metastatic Adenocarcinoma of the Pancreas. Eur J Cancer (2014) 50:2072–81. doi: 10.1016/j.ejca.2014.04.024 24915778

[71] da Cunha Santos G Dhani N Tu D Chin K Ludkovski O Kamel-Reid S. Molecular Predictors of Outcome in a Phase 3 Study of Gemcitabine and Erlotinib Therapy in Patients With Advanced Pancreatic Cancer: National Cancer Institute of Canada Clinical Trials Group Study PA.3. Cancer (2010) 116:5599–607. doi: 10.1002/cncr.25393 20824720

[72] Kullmann F Hartmann A Stöhr R Messmann H Dollinger MM Trojan J. KRAS Mutation in Metastatic Pancreatic Ductal Adenocarcinoma: Results of a Multicenter Phase II Study Evaluating Efficacy of Cetuximab Plus Gemcitabine/Oxaliplatin (GEMOXCET) in First-Line Therapy. Oncology (2011) 81:3–8. doi: 10.1159/000330194 21894049

[73] Daly MB Pilarski R Yurgelun MB Berry MP Buys SS Dickson P. NCCN Guidelines Insights: Genetic/Familial High-Risk Assessment: Breast, Ovarian, and Pancreatic, Version 1.2020. J Natl Compr Canc Netw (2020) 18:380–91. doi: 10.6004/jnccn.2020.0017 32259785

